# Dissolution of the Capitellum After Lateral Condylar Fracture of the Humerus in a Child: A Case Report

**DOI:** 10.7759/cureus.63975

**Published:** 2024-07-06

**Authors:** Kevin Newsome, Linda Qian, Monica Payares-Lizano

**Affiliations:** 1 Orthopedic Surgery, Herbert Wertheim College of Medicine, Florida International University, Miami, USA; 2 Orthopedic Surgery, Nicklaus Children's Hospital, Miami, USA

**Keywords:** capitellum, closed reduction percutaneous pinning, avascular necrosis, pediatric distal humerus fracture, lateral condyle fracture

## Abstract

Lateral condyle fractures of the humerus are a common elbow injury sustained by pediatric patients. Complications from surgical intervention can include malunion, fishtail deformity, osteonecrosis, and avascular necrosis (AVN). AVN of the capitellum is a rare complication of lateral condyle fractures with very few cases reported in the current literature. Here we report the rare case of dissolution of the capitellum following closed reduction and percutaneous pinning for a lateral condylar fracture of the humerus of a six-year-old child performed at an outside hospital that was subsequently managed at our academic pediatric level 1 trauma center. Other than a long-arm cast, no specific intervention was provided as the patient remained neurovascularly intact and improved clinically. Through careful follow-up and conservative management, the patient regained the full range of motion of the elbow and clinical resolution of the fracture. The single posterior blood supply of the capitellum likely contributes to the pathophysiology of this condition and further supports the methodology of avoiding posterior soft tissue stripping during surgical correction of distal humerus fractures. We conclude that the prognosis of this condition is favorable and can be managed by conservative treatment.

## Introduction

Fractures of the lateral condyle of the humerus are the second most common elbow fracture in the pediatric population, accounting for 12-20% of all pediatric elbow fractures [1]. These injuries typically occur as a result of a fall onto an outstretched hand during activities or sports [1]. Lateral condyle fractures can result in pain, swelling, ecchymosis, and limited range of motion of the elbow. Severe or displaced fractures require surgical intervention followed by a period of immobilization to regain the full range of motion and function of the elbow joint [1]. Complications from surgical intervention can include malunion, fishtail deformity, osteonecrosis, and avascular necrosis (AVN) [2]. Particularly, AVN can occur due to direct trauma or disruption of blood vessels during surgical intervention [3]. 

AVN of the distal humerus is a rare complication of lateral condyle fractures with very few cases reported in the current literature. The largest study investigating this topic was conducted by Shabtai et al. at a large level-1 tertiary pediatric hospital and identified only seven patients who developed AVN over the course of a 14-year period with an incidence of 1.4% [4]. Current literature emphasizes the importance of early diagnosis and subsequent treatment of lateral condyle fractures to prevent further complications such as permanent deformity and/or functional impairment [3]. 

Here we present a rare case of a six-year-old male with a lateral condyle fracture treated with closed reduction and percutaneous pinning that resulted in the dissolution of the capitellum. This is a rare outcome of the injury with very few reported cases in literature. This case highlights the significant clinical implications of a lateral condyle fracture and the current evidence regarding the management of the dissolution of the capitellum. This case report has been reported in line with the Surgical CAse REport (SCARE) criteria [5].

## Case presentation

A right-handed six-year-old Hispanic male presented to our pediatric fracture clinic for the follow-up of a right lateral condylar humerus fracture sustained from a fall two weeks prior (timeline is outlined in Table 1). 

**Table 1:** Timeline of the patient’s course.

Day	Description
Day 0	The patient sustains a fall, presents to an outside hospital, and is found to have a displaced right lateral condyle humerus fracture.
Day 1	Fracture is treated surgically with closed reduction and percutaneous pinning at the outside hospital.
Day 18	The patient presents to our fracture clinic. Pin sites were adjusted and transitioned to a long-arm cast.
Day 32	First follow-up at our orthopedic clinic. Imaging shows a healing fracture. The case and pins are removed.
Day 46	Second follow-up at our orthopedic clinic. Imaging shows continued healing of the fracture. The patient was prescribed physical therapy for a limited range of motion.
Day 95	Third follow-up at our orthopedic clinic. Imaging shows continued healing of the fracture. The patient was instructed to return to full activity.
Day 253	Fourth follow-up at our orthopedic clinic. Imaging shows a healed fracture. The capitellum had disappeared.
Day 342	Fifth follow-up at our orthopedic clinic. Imaging shows continued capitellum dissolution with signs of possible reossification in the region of the medial capitellum.

The patient initially presented to an outside hospital where he was found to have a displaced right lateral condylar humerus fracture and was treated surgically with closed reduction and percutaneous pinning. The parents had difficulty arranging a follow-up appointment with the outside hospital so they made an appointment to follow up at our institution’s fracture clinic. The patient had a history of a previous type 1 supracondylar humerus fracture of the opposite elbow that had resolved with long-arm casting one month prior, but otherwise had no significant past medical history. The patient had no other previous surgical history, was not taking any medications, and had no known drug allergies.

**Figure 1:**
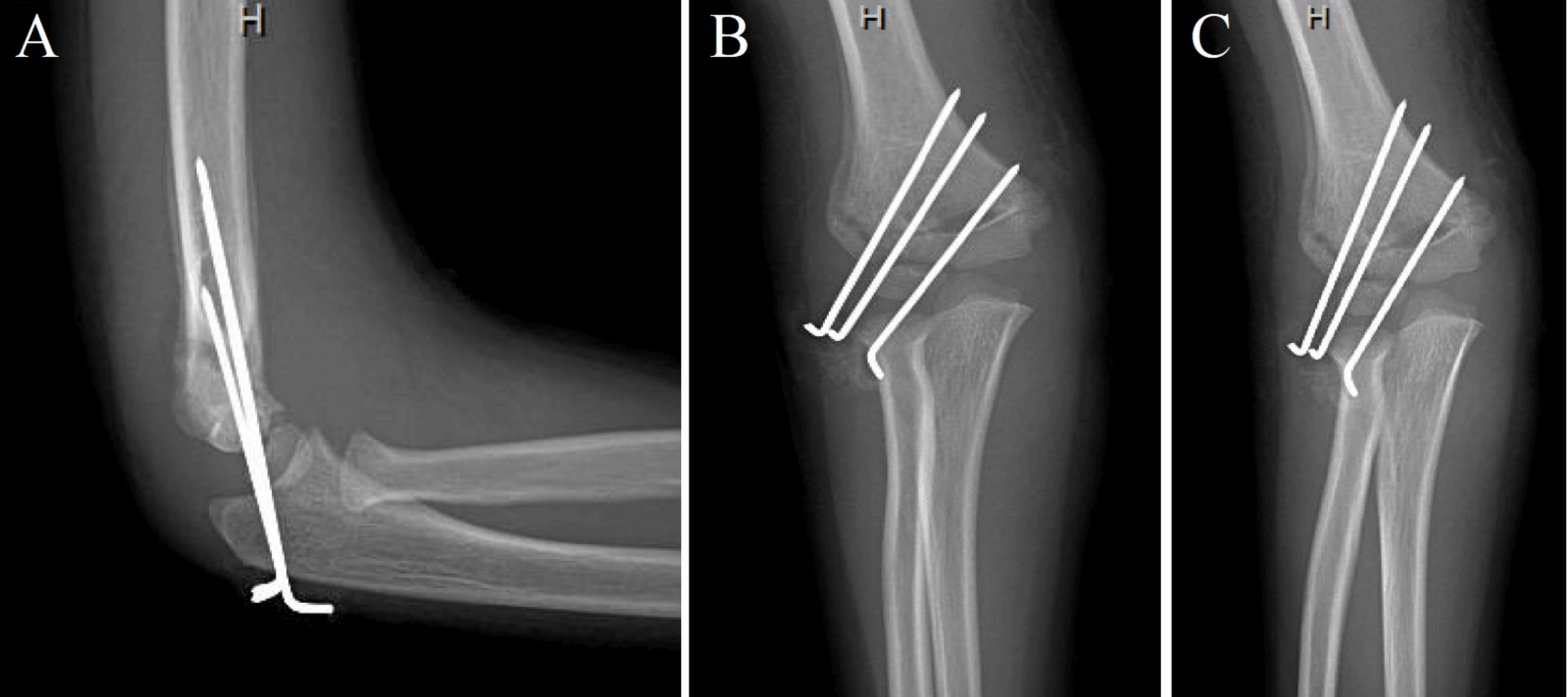
(A) Lateral, (B) anteroposterior, and (C) oblique X-ray views of the right elbow demonstrating the hardware-reduced condylar fracture with lateral pins and periosteal reaction and healing.

The patient had no complaints other than some irritation around the percutaneous pins and denied any fevers or chills. The arm was neurovascularly intact and X-ray imaging demonstrated some initial signs of healing with the fracture in good anatomic position (Figure 1). 

After removing the patient’s splint, the pins were found to be driven deeper inside and they were stuck to the skin, which was causing some irritation. There was evidence of granulation tissue, but no active signs of infection. The pins were pulled out slightly and were cleaned with peroxide. Some Xeroform was used to cover the pins and the arm was placed in a long-arm cast. The parents were hesitant to return to the original outside hospital for follow-up, so an appointment was made at our orthopedic clinic.

**Figure 2:**
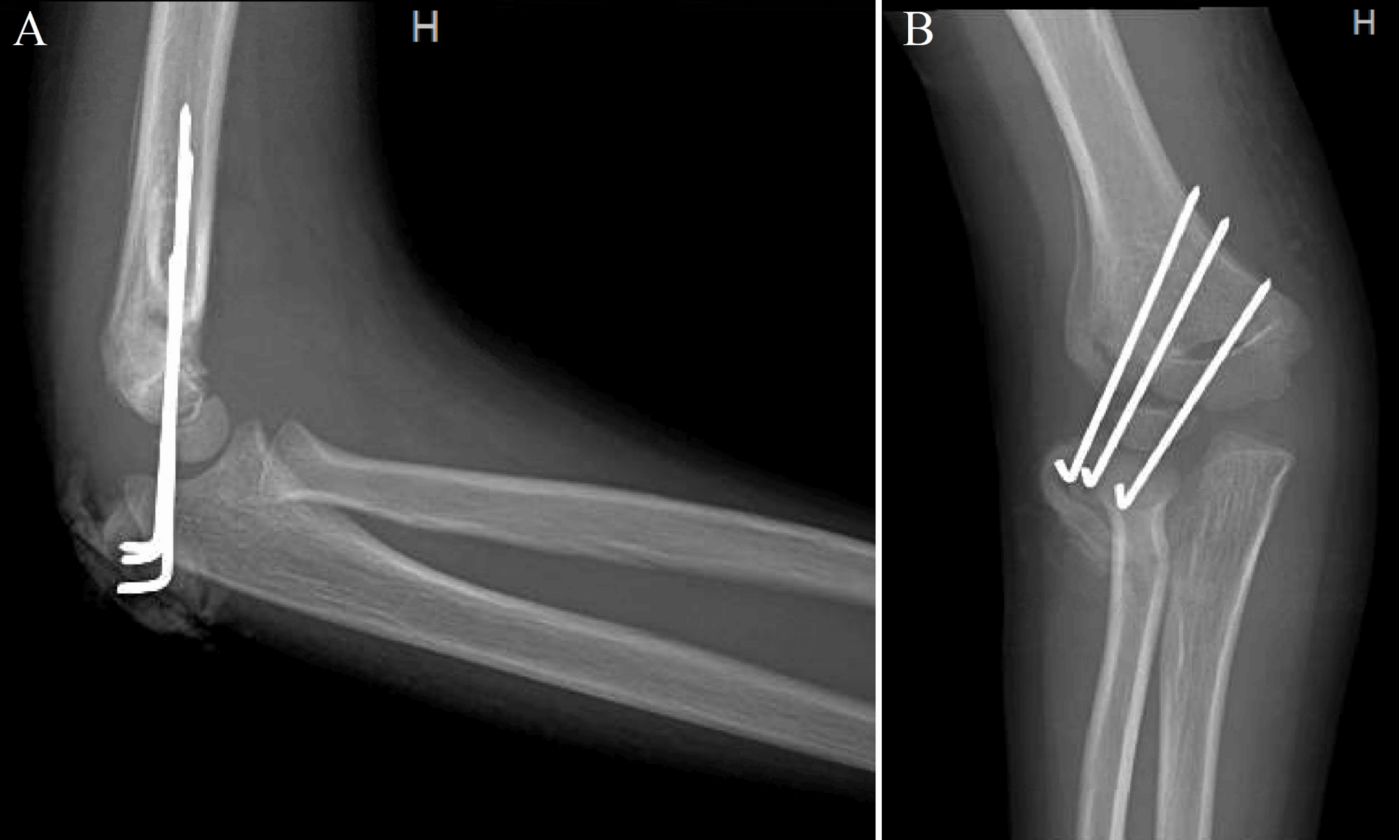
(A) Lateral and (B) anteroposterior X-ray views of the right elbow demonstrating progressive healing of the lateral condylar fracture in unchanged alignment.

On follow-up two weeks later, the arm remained neurovascularly intact and X-ray showed a healing fracture (Figure 2).

The cast was removed and there was continued irritation from the pins with the surrounding granulation tissue, but otherwise the site appeared to be healing well. The area was cleaned with peroxide and the pins were removed with some expected mild bleeding from the pin sites. The area was dressed with Xeroform and dry gauze. Four days after removing the pins, the patient presented to the emergency room for unrelated vomiting and coughing and tested positive for influenza A, but otherwise had no complaints. 

**Figure 3:**
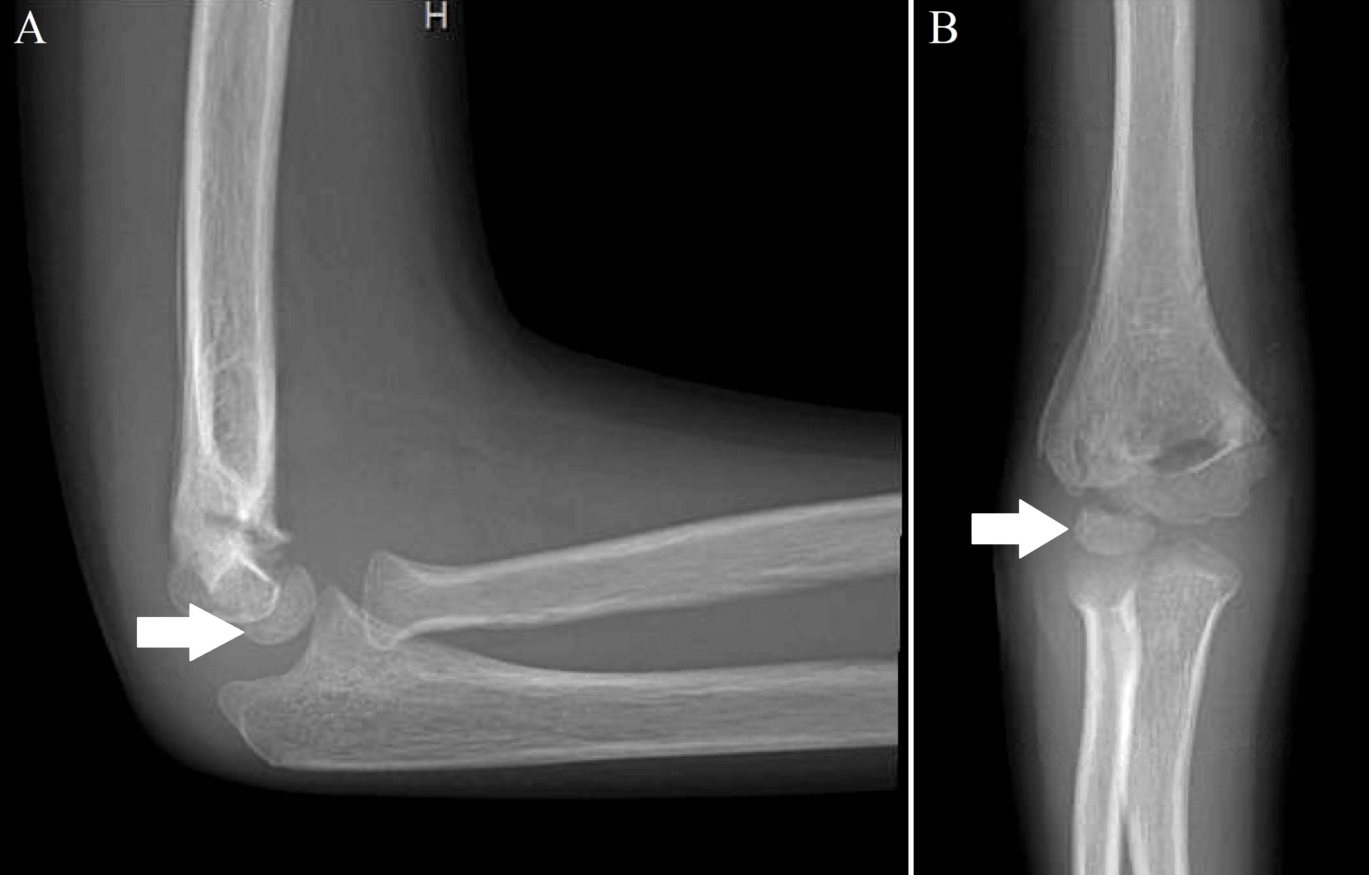
(A) Lateral and (B) anteroposterior X-ray views of the right elbow demonstrating hardware removal and further interval healing and remodeling of the supracondylar fracture with extension into the lateral condyle (the presence of intact capitellum is demonstrated by the arrow).

On the second follow-up four weeks later, the X-ray showed continued healing (Figure 3), but the patient complained of stiffness in his elbow. 

His passive and active range of motion (ROM) was limited to 100-120 degrees in the elbow and he was given a prescription for physical therapy and home exercises to improve ROM. On the third follow-up six weeks later, the patient’s ROM improved to full elbow flexion and neutral elbow extension. The X-ray showed continued healing. The patient was told to return to full activity. 

**Figure 4:**
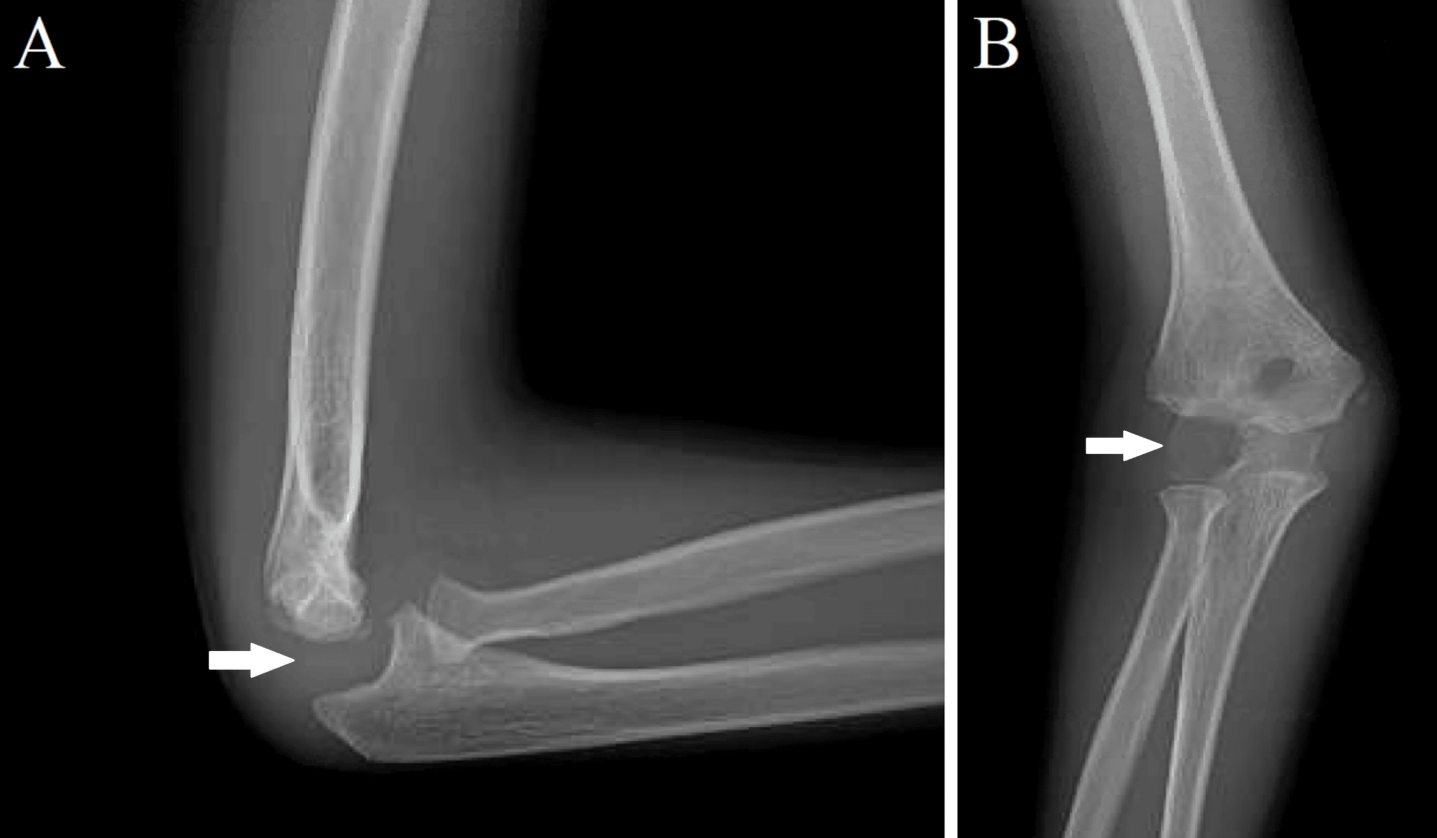
(A) Lateral and (B) anteroposterior X-ray views of the right elbow demonstrating the further late-healed appearance of the supracondylar fracture and interval loss of capitellum ossification with a ghost capitellum appearance (dissolution of the capitellum demonstrated by the arrow).

On the fourth follow-up four months later, the patient regained full ROM and had no complaints. The X-ray imaging showed a healed fracture; however, the capitellum had disappeared (Figure 4).

**Figure 5:**
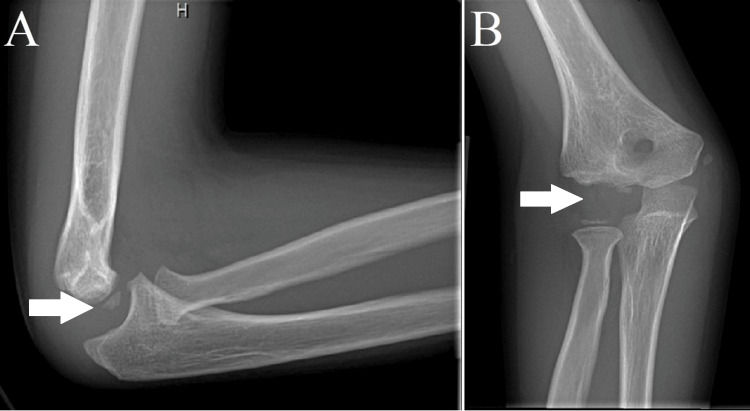
(A) Lateral and (B) anteroposterior X-ray views of the right elbow demonstrating a nearly healed lateral condylar fracture. The capitellum remains absent, however there is possible reossification in the medial aspect of the capitellar space (demonstrated by the arrow).

Considering the patient regained normal ROM and had normal strength and sensation in his elbow with no adverse symptoms, the patient was allowed to return to full activity. On the fifth follow-up three months later, a repeat X-ray showed that the capitellum remained absent; however, there were signs of possible reossification in the medial aspect of the capitellum region (Figure 5).

**Figure 6:**
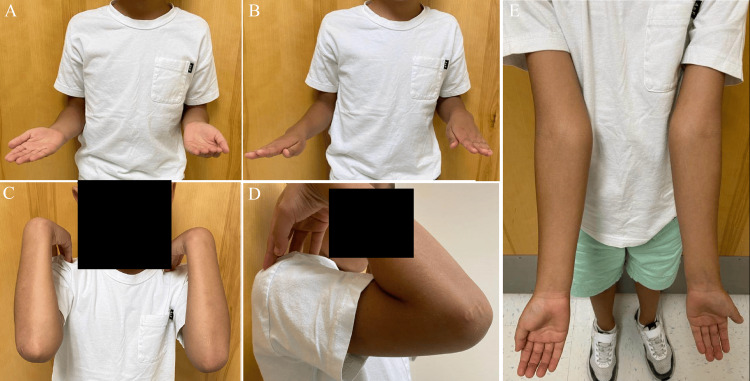
(A) Supination, (B) pronation, (C,D) flexion, and (E) extension of the patient’s arms demonstrating full and symmetrical range of motion of the right elbow during the fifth clinic visit.

The patient continued to have full and symmetric ROM of his right elbow and had no pain or neurovascular deficits (Figure 6). Further MRI imaging was deferred by the family due to out-of-pocket costs. 

## Discussion

Dissolution of the capitellum after lateral condyle fractures in pediatric patients has been reported sporadically throughout the literature. These instances appear to be most commonly associated with fractures that were treated by open reduction and percutaneous fixation. However, cases have been reported following conservative treatment with cast immobilization [4,6]. We reported a case of dissolution of the capitellum following closed reduction percutaneous pinning, a rare instance that has been reported very infrequently in the literature. 

The pathophysiology of dissolution of the capitellum following lateral condylar fractures is hypothesized to be related to the partial vascularization of the distal humerus [6]. Kimball et al. reported in an adult cadaveric study that there is a single vessel supplying the distal humerus with a predominantly posterior blood supply of the capitellar ossific nucleus [6,7]. This was supported by the findings of a cadaveric study by Yang et al., which mainly studied the microvascular supply of the capitellum of skeletally immature cadavers [8]. These findings suggest that injuries and disruption of the posterior capitellum may lead to compromised blood flow and subsequent demineralization of the capitellum. 

This explanation is consistent with the commonly taught approach to avoid posterior soft tissue stripping when performing surgery on the pediatric lateral condyle. Indeed a study by Wattenbarger et al. that investigated early versus late open reduction internal fixation (ORIF) of lateral condyle fractures on the incidence of AVN concluded that avoiding posterior soft tissue stripping during the surgical approach was more important than the timing of surgery to avoid AVN [9]. A study by Shabtai et al. noted that all of the cases of AVN that developed over the course of 14 years at their institution occurred following open reduction and percutaneous fixation. However, cases have been reported following a variety of interventions including conservative casting and immobilization [4,6]. A case has even been reported following an innovative arthroscopic-assisted reduction and percutaneous fixation of a lateral condyle fracture [10]. The authors of these studies all attribute the dissolution of the capitellum to potential posterior soft tissue stripping and recommend avoiding excessive damage to this area during fixation.

Previous studies have suggested that closed reduction percutaneous pinning is superior to ORIF in terms of rates of AVN, with the theoretical advantage of less soft tissue damage [11,12]. It is important to note, however, that AVN is exceptionally rare and these smaller studies may not represent a large enough sample size to adequately compare the complication rates of the techniques. Soft tissue destruction, regardless of technique, is also dependent on the skill of the surgeon and their familiarity with the procedure.

Generally, the outcome following the dissolution of the capitellum is favorable with the complete resolution being reported in a majority of the cases [4]. The most common complication is restricted ROM followed by valgus and varus deformities that can often be improved through conservative management and physical therapy. Capitellum remineralization is common on longer-term follow-up; however, in the absence of functional deficiencies, it is difficult to justify continued imaging and in-person examinations to confirm resolution, especially in the post-pandemic period. Based on the results of previous studies and the complete resolution of symptoms, it is reasonable that our patient’s capitellum will re-ossify spontaneously without the need for continued follow-up [6]. Other studies have suggested radiographs to be taken annually for five years followed by yearly clinical examinations. However, these patients likely receive little benefit from continued orthopedic care other than referrals from their primary physicians if there is clinical deterioration during routine examinations [6].

A strength of this case was the prolonged follow-up of nearly a full year after the initial injury. This allowed clinical and radiographic monitoring of the fracture both before and after dissolution of the capitellum, which provided a unique and longitudinal analysis of an extremely rare complication. The case was limited by the minimal information and imaging available from the patient's initial presentation to the outside hospital. 

Literature on this topic is difficult to analyze as the nomenclature used to refer to the dissolution of the capitellum varies significantly with terms such as radiolucency, demineralization, dissolution, disappearance, and avascular necrosis all being used interchangeably to refer to this phenomenon. Future studies may consider reaching a consensus regarding the nomenclature to improve consistency and comparability between studies.

## Conclusions

Here we reported the rare case of dissolution of the capitellum following closed reduction and percutaneous pinning for a lateral condylar fracture of the humerus in a six-year-old child. Through careful follow-up and conservative management, the patient regained full ROM of the elbow and clinical resolution of the fracture. The single posterior blood supply of the capitellum likely contributes to the pathophysiology of this condition and further supports the methodology of avoiding posterior soft tissue stripping during the surgical correction of distal humerus fractures. The prognosis of this condition is favorable and can be managed by conservative treatment.
